# An extracellular matrix paradox in myocardial scar formation

**DOI:** 10.1038/s41392-020-00270-z

**Published:** 2020-08-12

**Authors:** Sangkyun Cho, David T. Paik, Joseph C. Wu

**Affiliations:** 1 Stanford Cardiovascular Institute, Stanford University School of Medicine, Stanford, CA USA; 2 Department of Medicine, Division of Cardiovascular Medicine, Stanford University School of Medicine, Stanford, CA USA; 3 Department of Radiology, Stanford University School of Medicine, Stanford, CA USA

**Keywords:** Angiogenesis, Cardiology, Cell biology

**In a recent article in Cell**, **Yokota et al.**^1^ **demonstrate that type V collagen, a minor component of the cardiac extracellular matrix, plays a paradoxical role in limiting scar size after myocardial infarction by altering the mechanical properties of the developing scar tissue and by regulating integrin-dependent activation of cardiac fibroblasts** **(Fig.** **1****).**

**Fig. 1:**
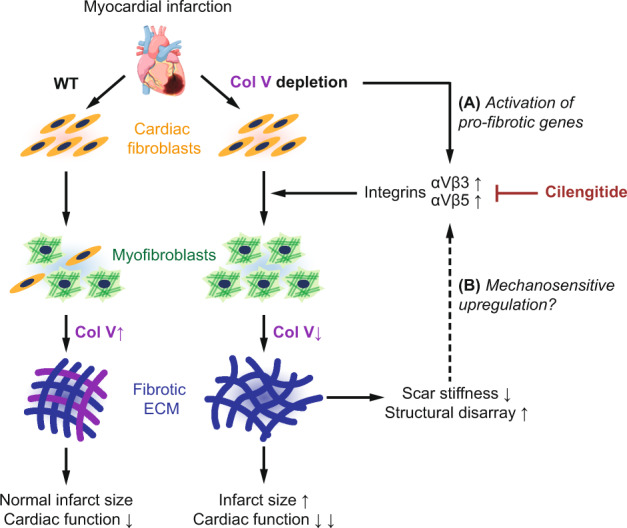
When less becomes more: Col V depletion results in a paradoxical increase in scar size post-MI. Type V collagen (Col V)-deficient mouse hearts subjected to experimental myocardial infarction (MI) exhibit increased scar size post-injury, with altered mechanical and structural properties. Col V-deficient fibroblasts exhibit elevated expression of integrins αVβ3 and αVβ5, which promote myofibroblast differentiation and could result from either **a** cell-intrinsic compensatory activation of pro-fibrotic gene programs, or **b** a feedback loop characterized by cell-extrinsic mechanosensitive upregulation, or both. Cilengitide, a specific inhibitor of αVβ3 and αVβ5 integrins, rescues the phenotype and suggests integrin-dependent signaling as a potential target for anti-fibrotic therapeutics

Upon myocardial infarction (MI), massive ischemic death of cardiomyocytes is replaced by a dense fibrous network of extracellular matrix (ECM), which holds the damaged tissue intact. The size of the resulting scar is a major predictor of patient outcomes and mortality, yet molecular mechanisms that regulate scar formation in the heart are poorly understood. A wide range of signals have been identified to activate cardiac fibroblasts and induce their differentiation into myofibroblasts, the cells responsible for secreting the fibrotic ECM. However, the precise functions of each ECM component deposited into the developing scar tissue remain unclear. Conventional wisdom suggests that a marked increase in ECM (fibrillar collagens in particular) would primarily serve to confer mechanical integrity and to stiffen the myocardium,^2^ promoting further differentiation of fibroblasts^3^ and ultimately resulting in a larger, more robust scar. However, a recent study by Yokota et al. suggests a minor component of the cardiac ECM, type V collagen (Col V), might be an exception to this paradigm.

To investigate the role of Col V in myocardial scar regulation, the authors first subjected mice to ischemic injury and performed transcriptional profiling at various time points to examine temporal changes in gene expression. RNA sequencing revealed highly dynamic transcriptional changes during the first week after MI but minimal differences beyond 2 weeks. As expected, collagens were among the most differentially expressed genes identified, with most isoforms exhibiting sharp increases between days 3 and 7. Among the fibrillar collagens, Col5a1 was found to be upregulated particularly early on, and this rapid increase in Col V was found to be expressed by the same fibroblasts that secrete type I and type III collagens, as confirmed by fluorescence in situ hybridization (FISH) and single-cell RNA sequencing (scRNA-seq).

Given the early upregulation of Col V following MI, the investigators then generated Col5a1 conditional knockout (CKO) mice to understand the functional consequence of Col V depletion. Col V-deficient mice exhibited significant reduction in cardiac performance by day 7, which persisted for the next 6 weeks. Histology further revealed a paradoxical increase in scar size in the Col5a1 CKO mice, the majority of which developed scars covering >40% of the total tissue. Col V depletion concomitantly resulted in altered cross-linking and structural rearrangement of collagen fibers, portending potential changes in the mechanical properties of the scar.

As a large number of ECM proteins including Col V were found to be upregulated simultaneously following MI, the investigators next examined the relative functional importance of Col V in driving post-injury cardiac function vis-à-vis other ECM genes. A hybrid mouse diversity panel of 100 strains were subjected to continuous isoproterenol infusion to induce chronic injury, and key cardiac functional metrics (e.g., contractility) were measured in relation to gene expression changes. Along with several other ECM genes, Col5a1 expression exhibited modest but statistically significant correlation with functional traits. Subsequent conditional analyses further revealed that correlations between ECM genes and cardiac functional traits (“ECM:traits”) were also largely dependent on Col5a1 expression, supporting the investigators’ central finding that Col V is a key driving factor in cardiac injury repair.

To understand the mechanisms by which Col V regulates scar formation and tissue repair, scRNA-seq was performed on non-myocyte cells in the control and Col5a1 CKO hearts. Analysis of differentially expressed genes revealed an increased fraction of Acta2+ myofibroblastic cells, as well as elevated expression of canonical myofibroblast genes (e.g., Acta2), ECM genes, and ECM crosslinkers (e.g., Lox) in the Col5a1 CKO heart. Fibroblast proliferation remained unaffected, suggesting that the reported increase in myofibroblasts is likely a direct result of enhanced differentiation of quiescent fibroblasts.

Given the significant increase in the number of myofibroblasts in the Col V-deficient scar, the investigators then hypothesized that altered scar mechanics could be an important factor driving myofibroblast differentiation. Surprisingly, atomic force microscopy measurements revealed that the Col V-depleted scars were less stiff versus control scars, contrary to the reported increase in myofibroblasts and the corresponding upregulation of ECM genes. The results also appear to be at odds with the suggested increase in collagen cross-linking (as assayed by insoluble collagen fraction and also implied by scRNA-seq data), a major determinant of tissue stiffness.^4^ At present, it is thus unclear how higher overall ECM expression and elevated cross-linking might give rise to a softer, more expansible scar. Nevertheless, cardiac fibroblasts isolated from the Col5a1 CKO scars did exhibit reduced contractility and deformability, indicative of possible perturbations in one or more mechanosensitive pathways.

To test the hypothesis that altered scar stiffness is indeed the driving factor for increased myofibroblast differentiation, the investigators generated Col5a1-deficient cardiac fibroblasts ex vivo. Myofibroblast genes as well as ECM genes were upregulated in the Col5a1-deficient fibroblasts, suggesting that Col V depletion is sufficient to initiate activation of a myofibroblast gene program perhaps by a compensatory mechanism. Flow cytometry and immunostaining further revealed that a significantly greater fraction of Col5a1-deficient cardiac fibroblasts expressed αVβ3 and αVβ5 integrins. Inhibition of these specific integrin isoforms by cilengitide, a cyclic RGD peptide, was sufficient to rescue the expression of myofibroblast genes. Importantly, cilengitide treatment in Col5a1 CKO mice after MI resulted in better preserved cardiac function, reduced fibrosis, and suppression of myofibroblast genes, suggesting αV integrins play a key role in myofibroblast differentiation and subsequent scar maturation.

Taken together, these studies suggest a previously unrecognized role for Col V in regulating scar size after MI. Combined with the cilengitide rescue experiments, the findings point to integrin-based mechanosensitive signaling as a potential target for future anti-fibrotic therapeutics, consistent with another recent report on an anti-integrin αV therapy in MI.^5^ However, although the phenotypic consequences of Col V deficiency seem apparent, whether the integrin-dependent activation of fibroblasts in this study can truly be attributed to the reduced stiffness of the Col V-depleted scar remains to be seen.

It would be intriguing to assess whether culturing normal cardiac fibroblasts on a Col V-deficient infarct scar would stimulate αV integrin expression and subsequent myofibroblast differentiation, and, conversely, whether inclusion of Col V in the ECM could rescue the phenotype of Col5a1-deficient fibroblasts. Similarly, fibroblast-specific gain-of-function of Col V in MI models will be necessary to assess the potentially beneficial role of Col V overexpression in modulation of myocardial scar formation. Such follow-up studies would be helpful in consolidating the mechanistic link between scar mechanics and cardiac myofibroblast differentiation. In conclusion, with a deeper understanding of causal mechanisms, the findings from the current study will likely have significant implications for treating fibrosis in a broad range of diseases in various organ systems in addition to the heart.

## References

[1] Yokota T (2020). Type V collagen in scar tissue regulates the size of scar after heart injury. Cell.

[2] Cho S (2019). Mechanosensing by the lamina protects against nuclear rupture, DNA damage, and cell-cycle arrest. Dev. Cell.

[3] Wipff PJ, Rifkin DB, Meister JJ, Hinz B (2007). Myofibroblast contraction activates latent TGF-beta1 from the extracellular matrix. J. Cell Biol..

[4] Frangogiannis NG (2017). The extracellular matrix in myocardial injury, repair, and remodeling. J. Clin. Investig..

[5] Bouvet M (2020). Anti-integrin αv therapy improves cardiac fibrosis after myocardial infarction by blunting cardiac PW1(+) stromal cells. Sci. Rep..

